# Influence of emotion on precision grip force control: A comparison of pleasant and neutral emotion

**DOI:** 10.3389/fpsyg.2022.1038522

**Published:** 2022-12-02

**Authors:** Yoshibumi Bunno, Chieko Onigata

**Affiliations:** ^1^Graduate School of Health Sciences, Graduate School of Kansai University of Health Sciences, Osaka, Japan; ^2^Clinical Physical Therapy Laboratory, Faculty of Health Sciences, Kansai University of Health Sciences, Osaka, Japan

**Keywords:** emotion, pleasant emotion, force steadiness, precision pinch grip, international affective picture system (IAPS)

## Abstract

**Objective:**

The present study aimed to investigate the impact of emotion on force steadiness of isometric precision pinch grip that is not direction-specific.

**Methods:**

Thirty-two healthy volunteer subjects participated in the present study. Subjects were divided into two experimental groups: pleasant image group and neutral image group. The isometric precision pinch grip task was performed for three times. Specifically, the first task was performed before pleasant or neutral picture viewing, the second task was performed immediately after picture viewing, further the third task was performed 30 seconds after the second task. During the isometric precision pinch grip task, participants were asked to exert pinch grip force at 10% of maximal voluntary contraction with visual feedback. The coefficient of variation of force production and normalized root mean square value of electromyography activity were calculated.

**Results:**

After pleasant picture viewing, coefficient of variation of pinch force production and normalized root mean square value of electromyography was decreased. While, in the neutral image condition, theses variables were not altered. More important, compared to the neutral image condition, pleasant emotion led to lower coefficient of variation of pinch grip force production.

**Conclusion:**

These findings indicate that pleasant emotion improves force control of isometric precision pinch grip. Therefore, in clinical settings, the emotional state of patients may affect the effectiveness of rehabilitation and should be taken into consideration.

## Introduction

Emotional experiences have a strong impact on motor behavior. Emotion is fundamentally organized according to valence (i.e., appetitive or defensive) and intensity (i.e., arousal) (Lang et al., 1998). The appetitive or defensive systems are engaged by external stimuli, and that affect preparation for action. In general, unpleasant emotions activate the defensive system, which facilitates avoidance movements away from stimuli. Whereas, pleasant emotions activate the appetitive system, which facilitates approach movements toward stimuli. The traditional view has been that motivational direction (i.e., approach or avoidance) and emotional valence (i.e., pleasant or unpleasant) are inextricably linked, however, it should be noted that elicited emotion (e.g., anger) and the motivational direction may not congruent (Elliot et al., 2013). Further, faster movement speed (Coombes et al., 2005) and greater force output (Coombes et al., 2006) have been associated with the activated defensive system. These previous investigations have conducted to reveal the relationship between the motivational direction and the given emotion elicited by external stimuli. However, many activities of daily living require precision pinch grip (e.g., buttoning a shirt, holding a coin, manipulating chopsticks) by thumb and index finger with isometric muscle contraction. Isometric precision pinch grip by thumb and index finger is non-directional movement. Thus, it should be important to consider the impact of emotion on motor behavior unrelated to movement direction.

When a person attempts to perform a steady muscle contraction, the force output is not constant and fluctuates about a given force level. Force steadiness is often quantified as the coefficient of variation (CV), a ratio of the standard deviation in force fluctuation relative to the mean force. A lower degree of CV indicates better force steadiness and greater force control. Similar with force output or speed, force steadiness can be influenced by emotional states. Unpleasant emotion evoked by a threat of electrical shock manipulation increased CV (Noteboom et al., 2001; Christou, 2005). In contrast, unpleasant emotion evoked by affective visual stimuli did not alter CV relative to neutral visual stimuli (Coombes et al., 2008; Naugle et al., 2012; Blakemore et al., 2016). As mentioned above, the impact of emotion on force steadiness is conflicting. However, Christou (2005) and Noteboom et al. (2001) evoked unpleasant emotion by using a threat of electrical shock. A threat shock manipulation may be more severe than visual stimuli using affective images. Therefore, the modality of affective stimuli may account for conflicting between previous studies (Noteboom et al., 2001; Christou, 2005; Coombes et al., 2008; Naugle et al., 2012; Blakemore et al., 2016).

Further, similar with unpleasant emotion, pleasant emotion evoked by visual stimuli did not alter CV (Coombes et al., 2008; Naugle et al., 2012; Blakemore et al., 2016). This indicate that emotional valence does not affect precision force control. However, previous studies (Noteboom et al., 2001; Christou, 2005; Coombes et al., 2008; Naugle et al., 2012; Blakemore et al., 2016) investigated force steadiness of precision pinch grip without visual feedback while viewing affective images. In other words, subjects in their previous studies performed picture viewing and precision pinch tasks simultaneously. It is plausible that decline of allocation of attentional resources for each task influenced the results of force steadiness in previous studies. In the present study, to investigate the impact of pleasant emotion on isometric precision pinch task with visual feedback, force steadiness was evaluated before and after affective visual stimuli. Specifically, the first evaluation was performed before affective visual stimuli, and the second evaluation was performed immediately after affective visual stimuli. In addition, to examine whether the sustained effect of emotion on force steadiness will be observed, the third evaluation was performed. Affective visual stimuli were not given between the second and third evaluation of force steadiness.

Also, precision force control is decreased with aging (Galganski et al., 1993; Laidlaw et al., 2000; Lindberg et al., 2009) and disease such as stroke (Lodha et al., 2010; Kang and Cauraugh, 2015). Kubota and Demura (2011) indicated that the non-dominant hand is inferior in the ability to properly adjust force exertion values to demand values than the dominant hand. Investigating the impact of emotion on precision pinch grip force control by the non-dominant hand in healthy subjects could provide important fundamental knowledge for future clinical application to the elderly and stroke patients. Thus, in the present study, participants performed an isometric precision pinch grip task by the non-dominant hand.

Amplitude of force fluctuations (i.e., CV) has been found to be greatest at low force level (e.g., 10% of maximal voluntary contraction) in hand motor task (Enoka et al., 2003). Force fluctuations also can be influenced by motor unit activity (Taylor et al., 2003; Farina and Negro, 2015). Specifically, large discharge rate variability of motor units (Laidlaw et al., 2000; Patten and Kamen, 2000) and motor unit synchronization (Yao et al., 2000) increase CV. In addition, motor unit synchronization increases amplitude of surface electromyography (EMG) (Yao et al., 2000). Zachry et al. (2005) indicated that reduced EMG amplitude has been associated with increased movement accuracy. Thus, the present study assessed force steadiness during isometric pinch grip at 10% maximal voluntary contraction (MVC) by using CV and surface EMG.

## Materials and methods

### Subjects

A priori sample size estimation was performed with G^*^Power version 3.1.9.4 (Faul et al., 2007). A total sample size of 28 was required to detect a significance level of 0.05 with power of 0.80 and effect size of 0.60 (medium-effect). Thus, 32 healthy volunteers [16 males, 16 females, age (mean ± SD) = 20.81 ± 0.47 years, range 20–22 years] participated in present study. To achieve the same male-female ratio, 16 male subjects were randomly divided into two image conditions, and 16 female subjects were randomly divided into two image conditions. Consequently, 8 male and 8 female subjects were assigned to each image condition, pleasant image condition [8 males, 8 females, age (mean ± SD) = 20.8 ± 0.45 years, range 20–21 years] and neutral image condition [8 males, 8 females, age (mean ± SD) = 20.9 ± 0.50 years, range 20–22 years], respectively. There was no statistical difference in age between the two conditions (Welch's *t*-test, *t* = 0.745, df = 29.6, 95% CI = −0.21 to 0.47, *p* = 0.462, *r* = 0.140). All subjects were determined to be right-handed with the Edinburgh Handedness Inventory (Oldfield, 1971). Written informed consent was obtained before participating in present study. The study was approved by the Research Ethics Committee at Kansai University of Health Sciences (Approval number: 20–34) and conducted in accordance with the Declaration of Helsinki.

### Experimental procedures

#### Pinch force and EMG recording apparatus

Pinch force value was measured using EMG recording software (Vital Recorder 2; Kissei Comtec Co., Ltd., Matsumoto, Japan). Surface EMG activity was measured using a telemetry EMG system (MQ-8; Kissei Comtec Co., Ltd., Matsumoto, Japan) and EMG recording software (Vital Recorder 2; Kissei Comtec Co., Ltd., Matsumoto, Japan). Surface EMG activity was measured from the abductor pollicis brevis (APB) muscle, which is the prime thumb muscle for opposition movement (Skoff, 1998). A pair of disposable Ag/AgCl electrodes (Blue Sensor N-00-S; Ambu A/S, Ballerup, Denmark) was attached over the left APB muscle with an inter-electrode distance of 15 mm. The skin was cleaned with an abrasive gel (Nuprep® Skin Prep Gel; Weaver and Company, Inc., Aurora, CO, USA) to maintain skin impedance <5 kΩ.

#### Emotion manipulation

Two digitized photographs were selected from the International Affective Picture System (IAPS; Lang et al., 2008). A pleasant picture was selected to be of higher valence and arousal [Puppies: 1,710; valence (mean ± SD), 8.34 ± 1.12; arousal (mean ± SD) 5.41 ± 2.34] than a neutral one [Mug: 7,009; valence (mean ± SD), 4.93 ± 1.00; arousal (mean ± SD), 3.01 ± 1.97]. These pictures were presented to the subjects as pleasant and neutral image conditions, respectively. After all pinch tasks, subjects rated the emotional valence of each picture using the Visual Analog Scale (VAS; 0 mm = unpleasant, 50 mm = neutral, 100 mm = pleasant).

#### Experimental protocol

**Figure 1:**
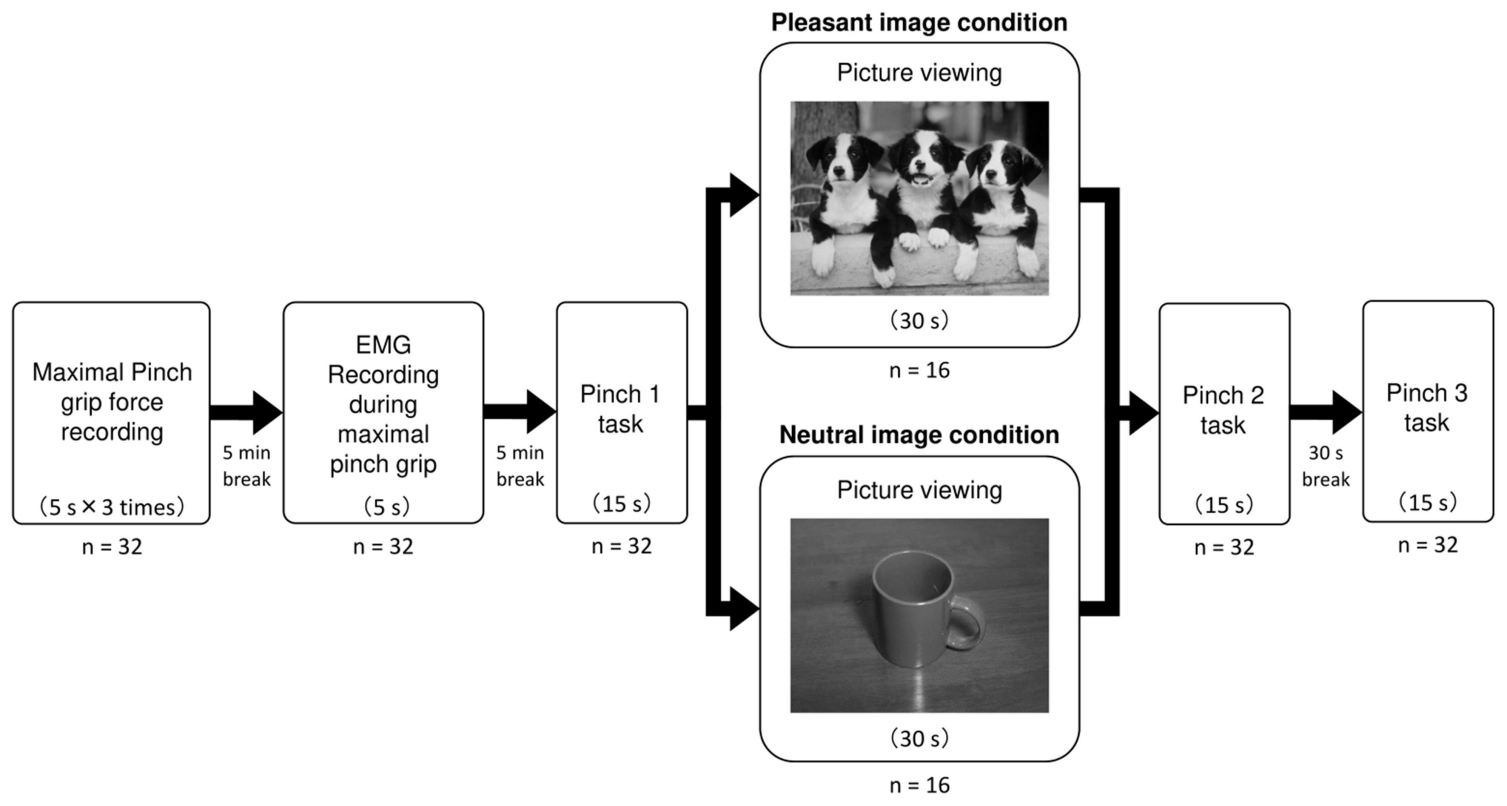
Experimental protocol. This figure shows experimental protocol in the present study. For each Pinch task, pinch force value and EMG activity were simultaneously recorded during precision pinch grip at 10% maximal pinch grip force. Subjects under the pleasant image condition viewed a pleasant picture of “Puppies”, while subjects under the neutral image condition viewed a neutral picture of “Mugs”.

Subjects were sitting comfortably a chair with their left arm resting on the armrest, and the forearm was fully supinated. Firstly, the maximal pinch grip force (kgf) recording was performed. The subjects pressed the sensor of the pinch meter (Digital indicator F340A; Unipulse Corp., Tokyo, Japan) between the left thumb and index finger with maximal effort for 5 s for three times. Maximal pinch grip force was determined as the mean maximal pinch grip force exerted for 5 s in each trial. After a 5 min break, EMG activity was measured while exerting the maximal pinch grip force for 5 s. After another 5 min break, subjects performed the first precision pinch grip task for one time (the Pinch 1 trial). Specifically, subjects were asked to exert their target level of force production (10% of maximal pinch grip force) as accurately as possible for 15 s while viewing the pinch force value numerically displayed on the pinch meter. Subsequently, subjects under the pleasant image condition viewed a pleasant picture (puppies) for 30 s, while subjects under the neutral image condition viewed a neutral picture (Mug) for 30 s. Each picture was projected on a 15.6 inches liquid crystal display (1,366 × 768 pixels resolution) of laptop computer (LIFEBOOK A573/GX, Fujitsu Ltd, Kanagawa, Japan) positioned 70 cm from their eyes. Immediately after picture viewing for 30 s, subjects performed the second precision pinch grip task for one time (the Pinch 2 trial). Followed by the pinch 2 trial, subjects rested for 30 s without viewing affective picture. And then, as with the Pinch 1 and 2 trial, subjects performed the third precision pinch grip task for one time (the Pinch 3 trial) (Figure 1).

### Data analysis

#### Pinch grip force

**Figure 2:**
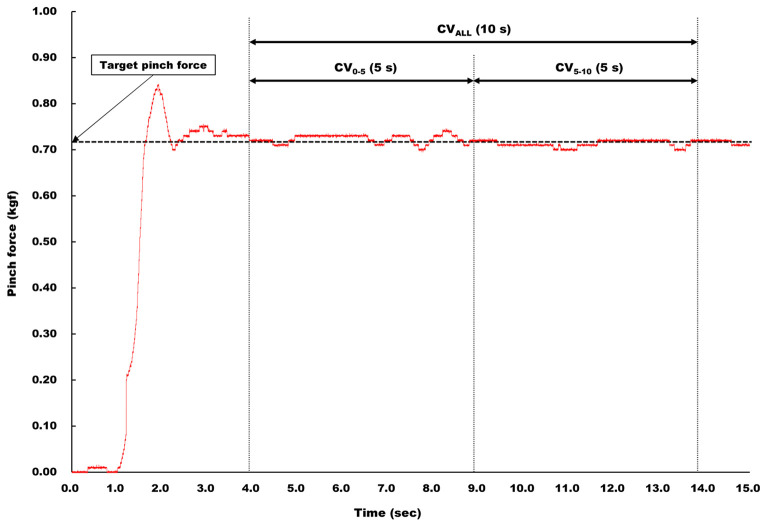
Analysis of force steadiness. Force steadiness was quantified as CV for three selected segments, termed CV_ALL_, CV_0 − 5_, and CV_5 − 10_, respectively, from the 10 s plateau of each pinch task. The duration of CV_ALL_ was 10 s, and that for CV_0 − 5_ and CV_5 − 10_ was 5 s.

The time series data of pinch grip force was sent to a laptop computer (LIFEBOOK A561/DX, Fujitsu Ltd., Kanagawa, Japan) *via* an A/D converter (ADA16-32/2(CB)F, Contec, Osaka, Japan) at a 1 kHz sampling rate with 16-bit resolution. First, to ensure the subjects successfully adjusted their target level of pinch grip force during an experiment, mean pinch grip force was calculated during each pinch trial. Force steadiness was quantified as the coefficient of variation [CV (%) = (SD/mean pinch grip force) × 100] for three selected segments, termed CV_ALL_, CV_0 − 5_, and CV_5 − 10_, respectively, from the 10 s plateau of each pinch task. The duration of segment for CV_ALL_ was 10 s, and that for CV_0 − 5_ and CV_5 − 10_ was 5 s (Figure 2).

#### Surface EMG activity

Raw surface EMG signals were filtered by a band-pass filter with a low-cut frequency of 10 Hz and a high-cut frequency of 1 kHz, and converted analog to digital at a 2 kHz sampling rate. The root mean square (RMS) value was calculated for three segments, termed RMS_ALL_, RMS_0 − 5_, and RMS_5 − 10_, respectively, the same as selected for CV (Figure 2). Obtained RMS values were normalized by RMS value of surface EMG during maximal pinch grip force production, and each normalized RMS value was expressed in %RMS_ALL_, %RMS_0 − 5_, and %RMS_5 − 10_, respectively.

### Statistical analysis

All variables were initially checked for normality by Shapiro-Wilk test. A parametric test was used for statistical analysis regarding the data confirmed normality, while for the data rejected normality, a non-parametric test was used. Particularly, for a parametric analysis of variance (ANOVA), Greenhouse-Geisser's epsilon correction was applied when Mendoza's multi-sample sphericity test suggested violations of the assumption of sphericity. Specific methods used for statistical analysis in the present study are described as follows.

#### Mean pinch grip force

To ensure whether subjects successfully exerted pinch grip force at their target level, mean pinch force values under each image (i.e., pleasant or neutral) condition were compared by using an ANOVA for repeated measures with factor of “trial” (i.e., Pinch 1, 2, and 3).

#### VAS score of the emotional valence

To ensure whether selected pictures evoked aimed emotion, VAS scores were compared between the pleasant and neutral conditions.

#### The influence of pinch task duration on force control

To examine the influence of real-time visual feedback on force control, CV_0 − 5_ and %RMS_0 − 5_ values were compared with CV_5 − 10_ and %RMS_5 − 10_ values for each trial under two image conditions.

#### The impact of emotion on force control

As described above, because no normality was found in some variables, we examined the impact of emotion on force control using following two analysis.

First, we examined whether pinch grip force control changes before and after affective visual stimuli. In each image condition, CV (CV_ALL_, CV_0 − 5_, and CV_5 − 10_) and %RMS (%RMS_ALL_, %RMS_0 − 5_, and %RMS_5 − 10_) values were compared by using an ANOVA for repeated measures with factor of “trial” (Pinch 1, 2, and 3). If the main effect of “trial” was confirmed, pairwise multiple comparison was performed for *post-hoc* analysis.

Second, we examined whether emotion-specific change will be observed in force control after viewing affective visual stimuli. Relative values were calculated under two image conditions by dividing the all CV (CV_ALL_, CV_0 − 5_, and CV_5 − 10_) and all %RMS (%RMS_ALL_, %RMS_0 − 5_, and %RMS_5 − 10_) values during the Pinch 1 trial with those during the Pinch 2 and 3 trials. Obtained relative values were compared between the pleasant and neutral image conditions.

The threshold for statistical significance was set at α = 0.05. Statistical analysis was performed using R commander (ver. 2.7.0) and R (ver. 4.0.2; R Core Team, 2020). An effect size was calculated in all significant data by R commander (ver. 2.7.0) and R (ver. 4.0.2; R Core Team, 2020).

## Results

First, the data (mean ± SD) for all variables for each trial under the two image conditions are listed in Tables 1–4.

**Table 1:** Mean pinch force values in the pleasant and neutral image conditions.

	**Pinch 1 trial**	**Pinch 2 trial**	**Pinch 3 trial**
**Pleasant image condition**			
Mean pinch force (kgf)	0.25 ± 0.08	0.25 ± 0.08	0.25 ± 0.07
**Neutral image condition**			
Mean pinch force (kgf)	0.23 ± 0.07	0.24 ± 0.07	0.24 ± 0.07

Mean ± SD.

**Table 2:** VAS scores in the pleasant and neutral image conditions.

	**Pleasant image condition**	**Neutral image condition**
VAS score (cm)	8.2 ± 1.1	5.2 ± 0.8

Mean ± SD.

**Table 3:** CV and %RMS values in the pleasant and neutral image conditions.

	**Pinch 1 trial**	**Pinch 2 trial**	**Pinch 3 trial**
**Pleasant image condition**			
CV_ALL_ value (%)	3.86 ± 1.65	3.47 ± 1.41	2.92 ± 0.84
CV_0 − 5_ value (%)	4.33 ± 1.84	3.93 ± 2.00	2.94 ± 0.80
CV_5 − 10_ value (%)	3.40 ± 1.81	3.00 ± 1.17	2.90 ± 1.25
%RMS_ALL_ value	0.169 ± 0.091	0.150 ± 0.099	0.130 ± 0.097
%RMS_0 − 5_ value	0.169 ± 0.088	0.148 ± 0.099	0.129 ± 0.095
%RMS_5 − 10_ value	0.171 ± 0.094	0.152 ± 0.101	0.130 ± 0.099
**Neutral image condition**			
CV_ALL_ value (%)	3.59 ± 1.41	3.44 ± 1.21	3.31 ± 1.14
CV_0 − 5_ value (%)	3.98 ± 1.79	3.93 ± 1.50	3.58 ± 1.19
CV_5 − 10_ value (%)	3.20 ± 1.27	2.96 ± 1.05	3.05 ± 1.51
%RMS_ALL_ value	0.169 ± 0.089	0.160 ± 0.055	0.142 ± 0.056
%RMS_0 − 5_ value	0.171 ± 0.088	0.162 ± 0.057	0.143 ± 0.057
%RMS_5 − 10_ value	0.168 ± 0.091	0.157 ± 0.057	0.141 ± 0.056

Mean ± SD.

CV, Coefficient of Variation; %RMS, %Root Mean Square.

**Table 4:** Relative values of CV and %RMS in the pleasant and neutral image conditions.

	**Pleasant image condition**	**Neutral image condition**
**Relative value of CV**		
Relative CV_0 − 5_ of pinch 2-to-1	1.02 ± 0.65	1.04 ± 0.31
Relative CV_0 − 5_ of pinch 3-to-1	0.74 ± 0.23	0.98 ± 0.33
Relative CV_5 − 10_ of pinch 2-to-1	0.96 ± 0.31	0.97 ± 0.25
Relative CV_5 − 10_ of pinch 3-to-1	0.98 ± 0.45	0.99 ± 0.34
**Relative value of %RMS**		
Relative %RMS_0 − 5_ of pinch 2-to-1	0.88 ± 0.30	1.05 ± 0.30
Relative %RMS_0 − 5_ of pinch 3-to-1	0.77 ± 0.28	0.92 ± 0.22
Relative %RMS_5 − 10_ of pinch 2-to-1	0.91 ± 0.29	1.07 ± 0.38
Relative %RMS_5 − 10_ of pinch 3-to-1	0.79 ± 0.31	0.95 ± 0.28

Mean ± SD.

CV, Coefficient of Variation; %RMS, %Root Mean Square.

### Mean pinch grip force

There were no significant differences in mean pinch grip force among pinch trials for both image conditions (Pleasant image condition: one-way repeated measures ANOVA, *F* (2, 30) = 2.45, *p* = 0.103, $\eta_{G}^{2}$ = 0.0001; Neutral image condition: one-way repeated measures ANOVA, *F* (1.18, 17.69) = 2.85, *p* = 0.105, $\eta_{G}^{2}$ = 0.0005, Table 1).

### Rate of emotional valence

**Figure 3:**
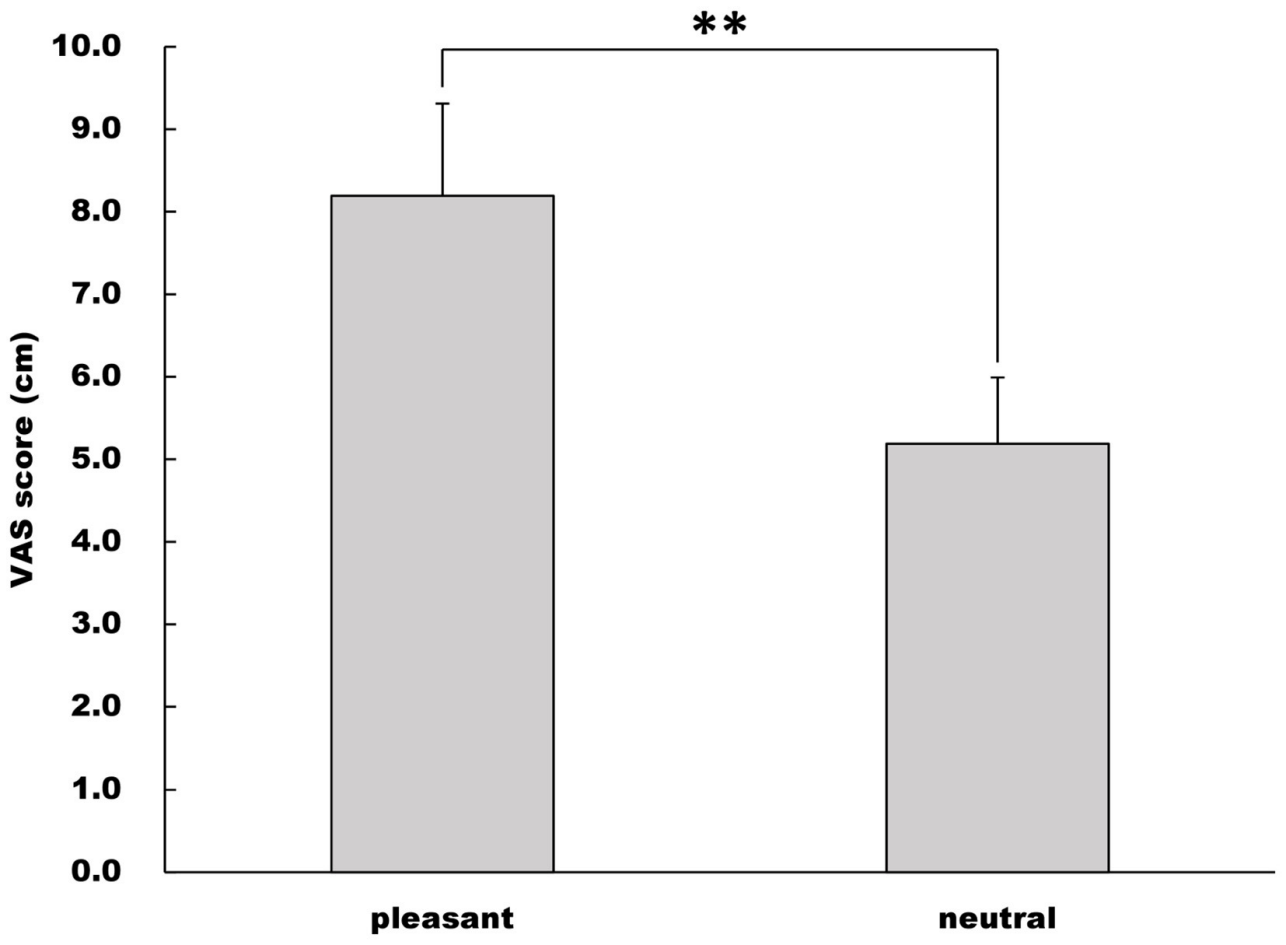
Rate of emotional valence. Emotional valence was quantified using the VAS score (cm). This figure shows the mean and standard deviation of VAS scores in the pleasant and neutral image conditions. **p < 0.01.

The VAS score under the pleasant image condition was higher than that under the neutral image condition (Welch's *t*-test, *t* = −8.74, df = 27.3, 95% CI = −3.71 to −2.30, *p* < 0.001, *r* = 0.860, Figure 3).

### Influence of pinch task duration on force control

**Figure 4:**
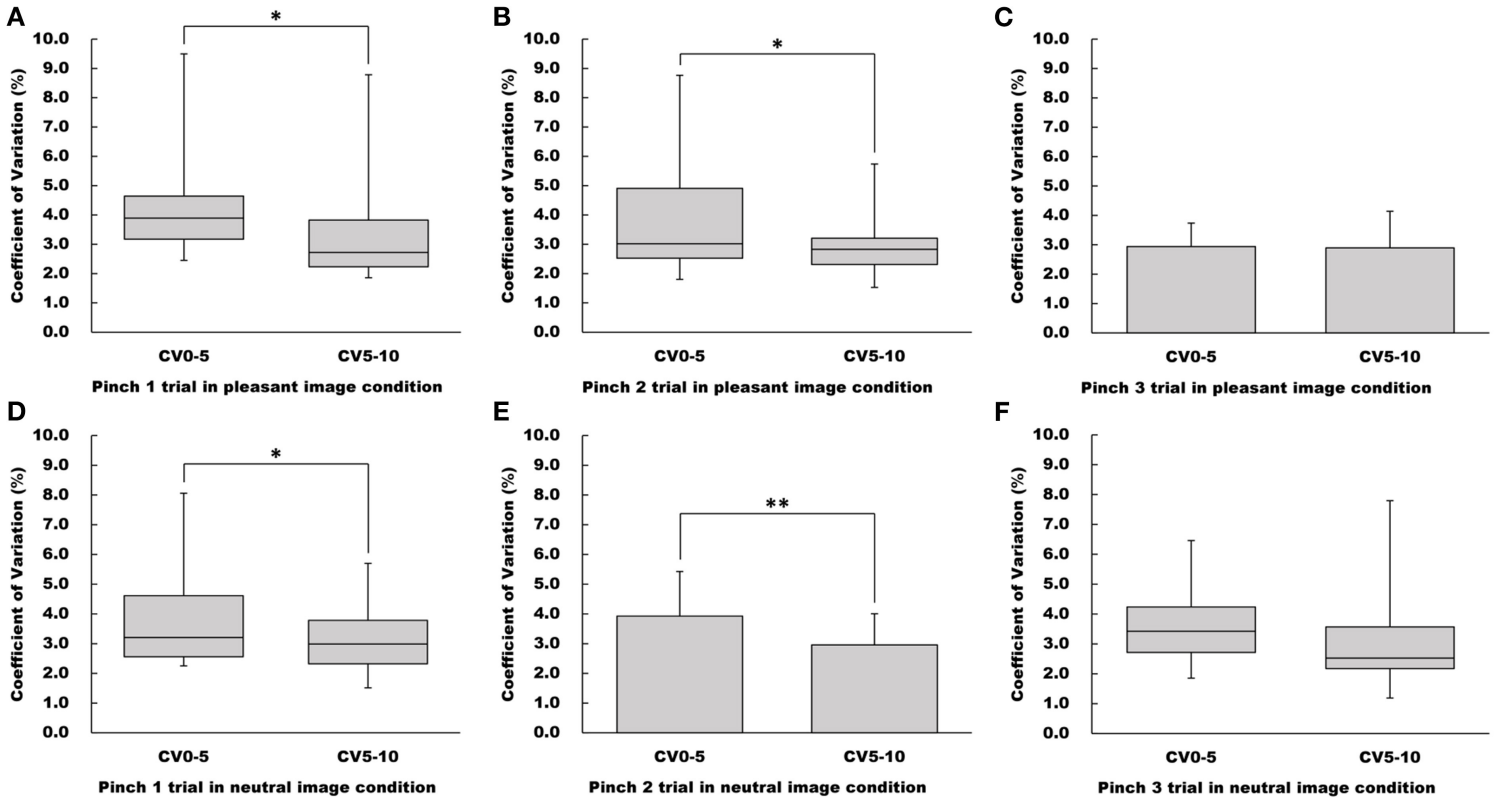
Influence of duration on CV values in the pleasant and neutral image conditions. **(A–C)** Show CV value in the pleasant image condition, respectfully. **(D–F)** Show CV value in the neutral image condition, respectfully. For parametric data, CV values are represented as mean and standard deviation. For non-parametric data, CV values are represented using the box and whisker plot, with the median shown as the middle horizontal line, the upper and lower quartiles indicated as boxes, and the maximum and minimum indicated as whiskers. The vertical axis shows CV value (%), and horizontal axis shows the specific duration (CV_0 − 5_ and CV_5 − 10_). *p < 0.05, **p < 0.01.

CV_5 − 10_ values for both the Pinch 1 and 2 trials under the pleasant image condition were significantly lower than the CV_0 − 5_ values (Pinch 1 trial: Wilcoxon signed-rank test, *Z* = −2.22, *p* < 0.05, *r* = 0.393; Pinch 2 trial: Wilcoxon signed-rank test, *Z* = −1.97, *p* < 0.05, *r* = 0.347, Figure 4). However, there was no significant difference between CV_0 − 5_ and CV_5 − 10_ values for the Pinch 3 trial under the pleasant image condition (Paired *t*-test, *t* = 0.138, 95% CI = −0.627 to 0.714, *p* = 0.892, *r* = 0.0356, Figure 4).

Regarding the neutral image condition, CV_5 − 10_ values for both the pinch1 and 2 trials were significantly lower than the CV_0 − 5_ values (Pinch 1 trial: Wilcoxon signed-rank test, *Z* = −1.97, *p* < 0.05, *r* = 0.347; Pinch 2 trial: Paired *t*-test, *t* = 4.11, 95% CI = 0.466 to 1.47, *p* < 0.001, *r* = 0.728, Figure 4). As observed in pleasant image condition, there was no difference between CV_5 − 10_ and CV_0 − 5_ values for the Pinch 3 trial (Wilcoxon signed-rank test, *Z* = −1.55, *p* = 0.121, *r* = 0.274, Figure 4).

**Figure 5:**
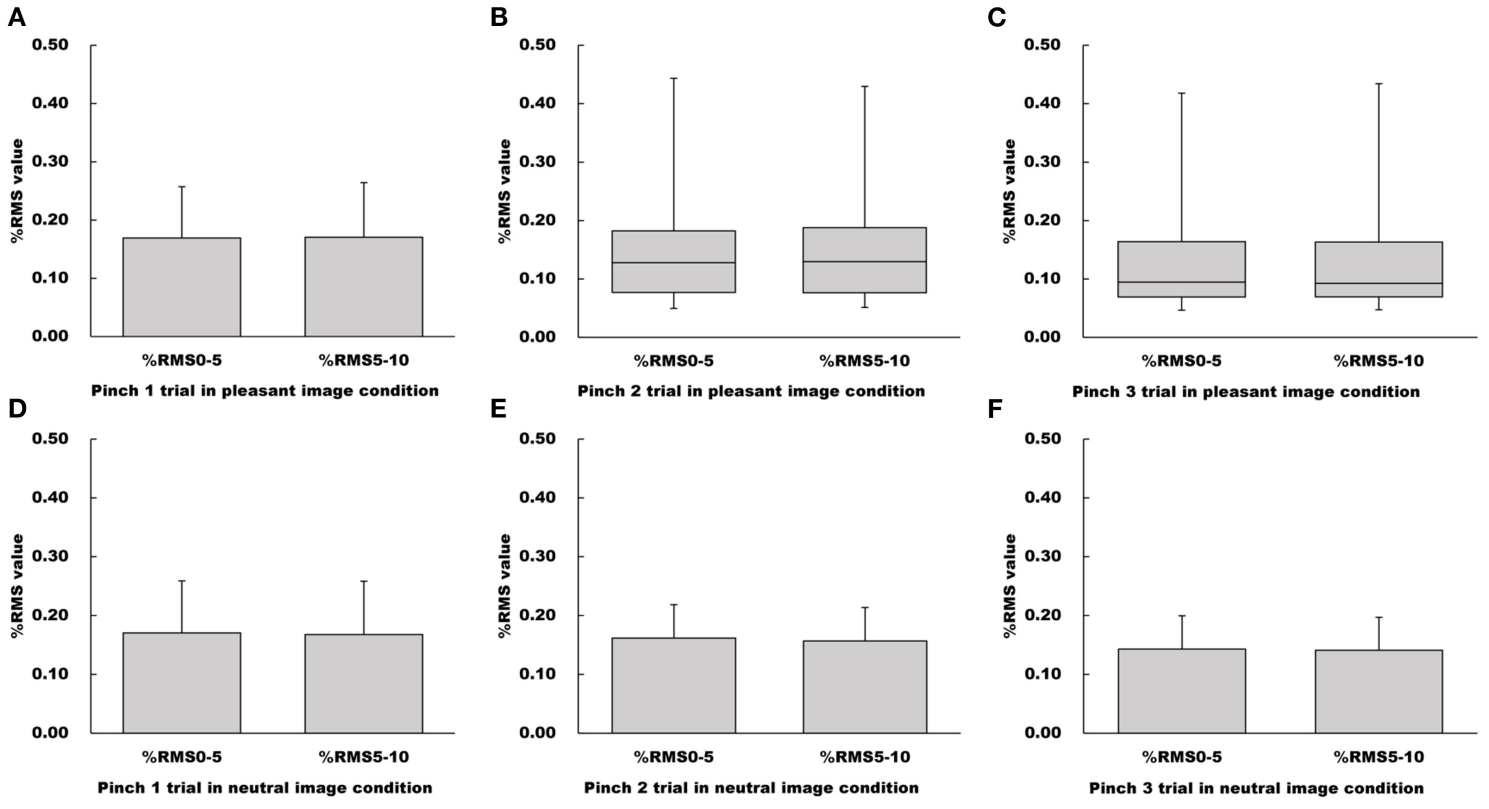
Influence of duration on %RMS values in the pleasant and neutral image conditions. **(A–C)** Show %RMS value in the pleasant image condition, respectfully. **(D–F)** Show %RMS value in the neutral image condition, respectfully. For parametric data, %RMS values are represented as mean and standard deviation. For non-parametric data, %RMS values are represented using the box and whisker plot, with the median shown as the middle horizontal line, the upper and lower quartiles indicated as boxes, and the maximum and minimum indicated as whiskers. The vertical axis shows %RMS value, and horizontal axis shows the specific duration (%RMS_0 − 5_ and %RMS_5 − 10_).

There were no differences between %RMS_5 − 10_ and %RMS_0 − 5_ values for all pinch tasks under the pleasant image condition (Pinch 1 trial: Paired *t*-test, *t* = −0.391, df = 15, 95% CI = −0.00928 to 0.00640, *p* = 0.702, *r* = 0.100; Pinch 2 trial: Wilcoxon signed-rank test, *Z* = −0.350, *p* = 0.726, *r* = 0.0620; Pinch 3 trial: Wilcoxon singed-rank test, *Z* = −0.441, *p* = 0.659, *r* = 0.0780, Figure 5).

Similarly, there were no significant differences between %RMS_5 − 10_ and %RMS_0 − 5_ values for all pinch tasks under the neutral image condition (Pinch 1 trial: Paired *t*-test, *t* = 0.481, df = 15, 95% CI = −0.00964 to 0.0153, *p* = 0.637, *r* = 0.123; Pinch 2 trial: Paired *t*-test, *t* = 0.677, df = 15, 95% CI = −0.0103 to 0.0120, *p* = 0.509, *r* = 0.172; Pinch 3 trial, Paired *t*-test, *t* = 0.666, df = 15, 95% CI = −0.00440 to 0.00840, *p* = 0.516, *r* = 0.169, Figure 5).

### Changes in pinch force control after the pleasant or neutral visual stimuli

**Figure 6:**
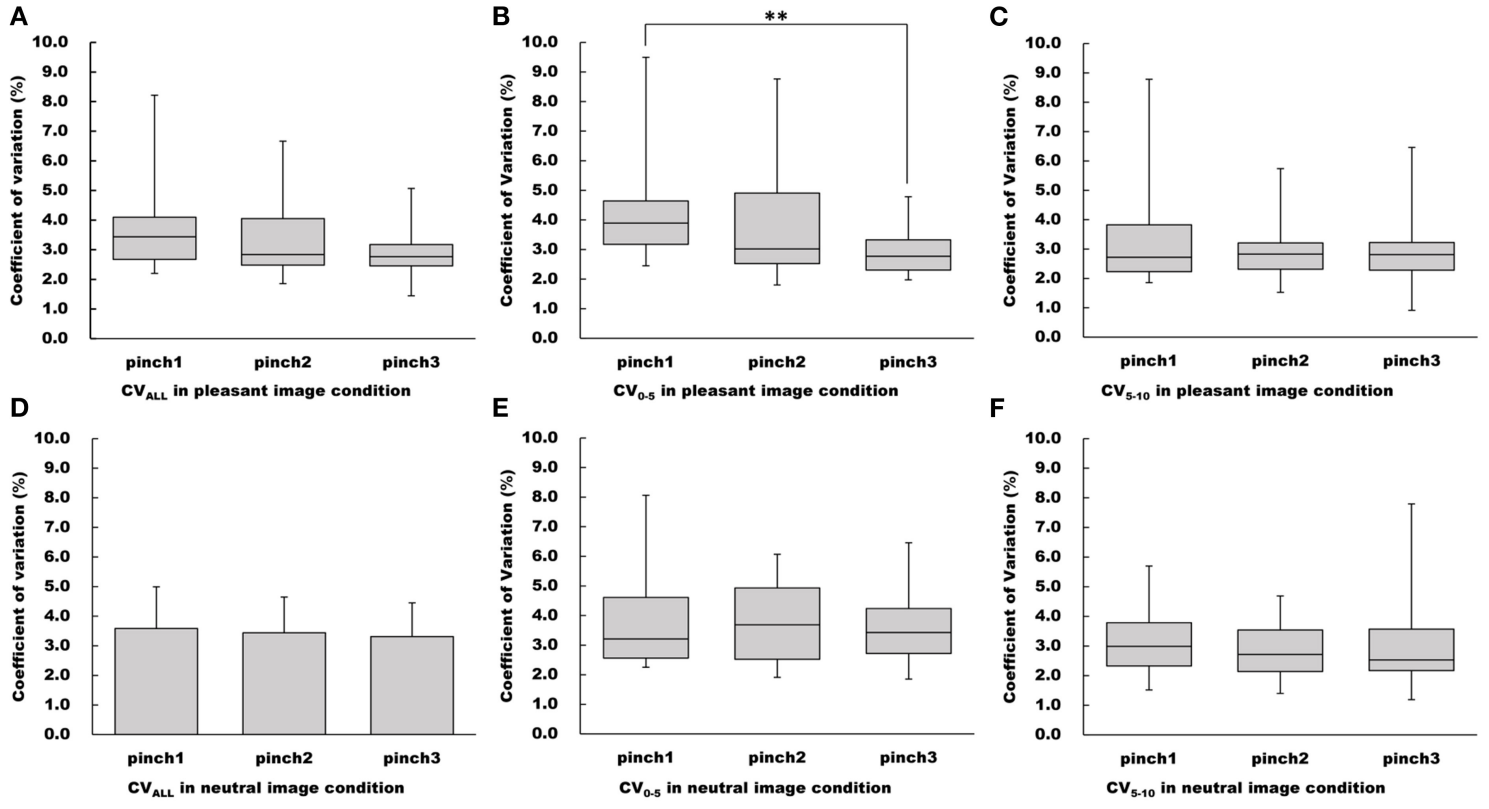
Changes of CV values in the pleasant and neutral image conditions. **(A–C)** Show CV_ALL_, CV_0 − 5_, CV_5 − 10_ values in the pleasant image condition, respectfully. **(D–F)** Show CV_ALL_, CV_0 − 5_, CV_5 − 10_ values in the neutral image condition, respectfully. For parametric data, CV values are represented as mean and standard deviation. For non-parametric data, CV values are represented using the box and whisker plot, with the median shown as the middle horizontal line, the upper and lower quartiles indicated as boxes, and the maximum and minimum indicated as whiskers. The vertical axis shows CV value (%), and horizontal axis shows the specific trial (Pinch 1, 2, and 3). **p < 0.01.

There was no main effect of “trial” on CV_ALL_ for the pleasant image condition (Friedman's test, chi-squared = 1.63, df = 2, *p* = 0.444, Figure 6). Whereas, the main effect of “trial” on CV_0 − 5_ for the pleasant image condition was confirmed (Friedman's test, chi-squared = 7.13, df = 2, *p* < 0.05, Figure 6). Furthermore, pairwise multiple comparison revealed that CV_0 − 5_ during the Pinch 3 trial was significantly lower than that during the Pinch 1 trial (Wilcoxon signed-rank test corrected with Holm's procedure, *p* < 0.01, *r* = 0.789, Figure 6). However, no main effect of “trial” on CV_5 − 10_ was confirmed for the pleasant image condition (Friedman's test, chi-squared = 1.50, df = 2, *p* = 0.472, Figure 6).

For the neutral image condition, no main effects of “trial” on CV_ALL_, CV_0 − 5_, and CV_5 − 10_ values were confirmed (CV_ALL_: one-way repeated measures ANOVA, *F* (2, 30) = 0.801, *p* = 0.458, $\eta_{G}^{2}$ = 0.0084; CV_0 − 5_: Friedman's test, chi-squared = 1.63, df = 2, *p* = 0.444; CV_5 − 10_: Friedman's test, chi-squared = 3.50, df = 2, *p* = 0.174, Figure 6).

**Figure 7:**
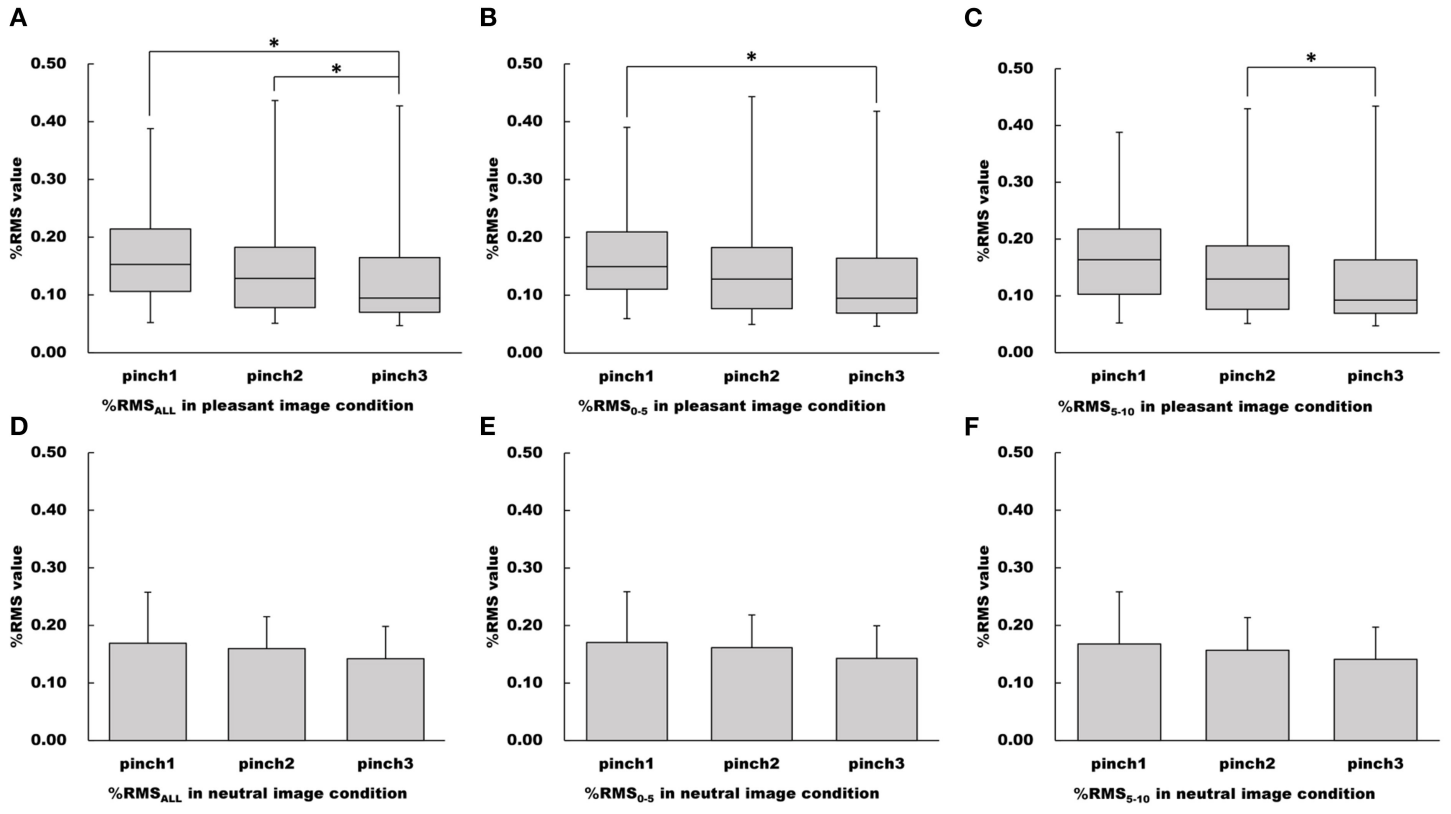
Changes of %RMS values in the pleasant and neutral image conditions. **(A–C)** Show %RMS_ALL_, %RMS_0 − 5_, %RMS_5 − 10_ values in the pleasant image condition, respectfully. **(D–F)** Show %RMS_ALL_, %RMS_0 − 5_, %RMS_5 − 10_ values in the neutral image condition, respectfully. For parametric data, %RMS values are represented as mean and standard deviation. For non-parametric data, %RMS values are represented using the box and whisker plot, with the median shown as the middle horizontal line, the upper and lower quartiles indicated as boxes, and the maximum and minimum indicated as whiskers. The vertical axis shows %RMS, and horizontal axis shows the specific trial (Pinch 1, 2, and 3). *p < 0.05.

The main effect of “trial” on the %RMS_ALL_ value was confirmed for the pleasant image condition (Friedman's test, chi-squared = 8.10, df = 2, *p* < 0.05, Figure 7). Pairwise multiple comparison for the pleasant image condition revealed that the %RMS_ALL_ value during the Pinch 3 trial was significantly lower than that during the Pinch 1 and 2 trials (Pinch 3 trial vs. Pinch 1 trial: Wilcoxon signed-rank test corrected with Holm's procedure, *p* < 0.05, *r* = 0.568; Pinch 3 trial vs. Pinch 2 trial: Wilcoxon signed-rank test corrected with Holm's procedure, *p* < 0.05, *r* = 0.673, Figure 7).

Additionally, the main effect of “trial” on the %RMS_0 − 5_ value for the pleasant image condition was confirmed (Friedman's test, chi-squared = 6.10, df = 2, *p* < 0.05, Figure 7). Pairwise multiple comparison revealed that the %RMS_0 − 5_ value during the Pinch 3 trial was significantly lower than that during the Pinch 1 trial (Wilcoxon signed-rank test corrected with Holm's procedure, *p* < 0.05, *r* = 0.625, Figure 7). Moreover, the main effects of “trial” on the %RMS_5 − 10_ value for pleasant image condition were confirmed (Friedman's test, chi-squared = 8.32, df = 2, *p* < 0.05, Figure 7). Pairwise multiple comparison revealed that the %RMS_5 − 10_ value during the Pinch 3 trial was significantly lower than that during the Pinch 2 trial (Wilcoxon signed-rank test corrected with Holm's procedure, *p* < 0.05, *r* = 0.620, Figure 7).

For the neutral image condition, the main effects of “trial” on the %RMS_ALL_, %RMS_0 − 5_, and %RMS_5 − 10_ values were not confirmed (%RMS_ALL_: one-way repeated measures ANOVA, *F* (2, 30) = 2.20, *p* = 0.129, $\eta_{G}^{2}$ = 0.0274; %RMS_0 − 5_: one-way repeated measures ANOVA, *F* (2, 30) = 2.32, *p* = 0.116, $\eta_{G}^{2}$ = 0.0286; %RMS_5 − 10_: one-way repeated measures ANOVA, *F* (2, 30) = 1.94, *p* = 0.162, $\eta_{G}^{2}$ = 0.0257, Figure 7).

### Influence of difference in emotion on force control

**Figure 8:**
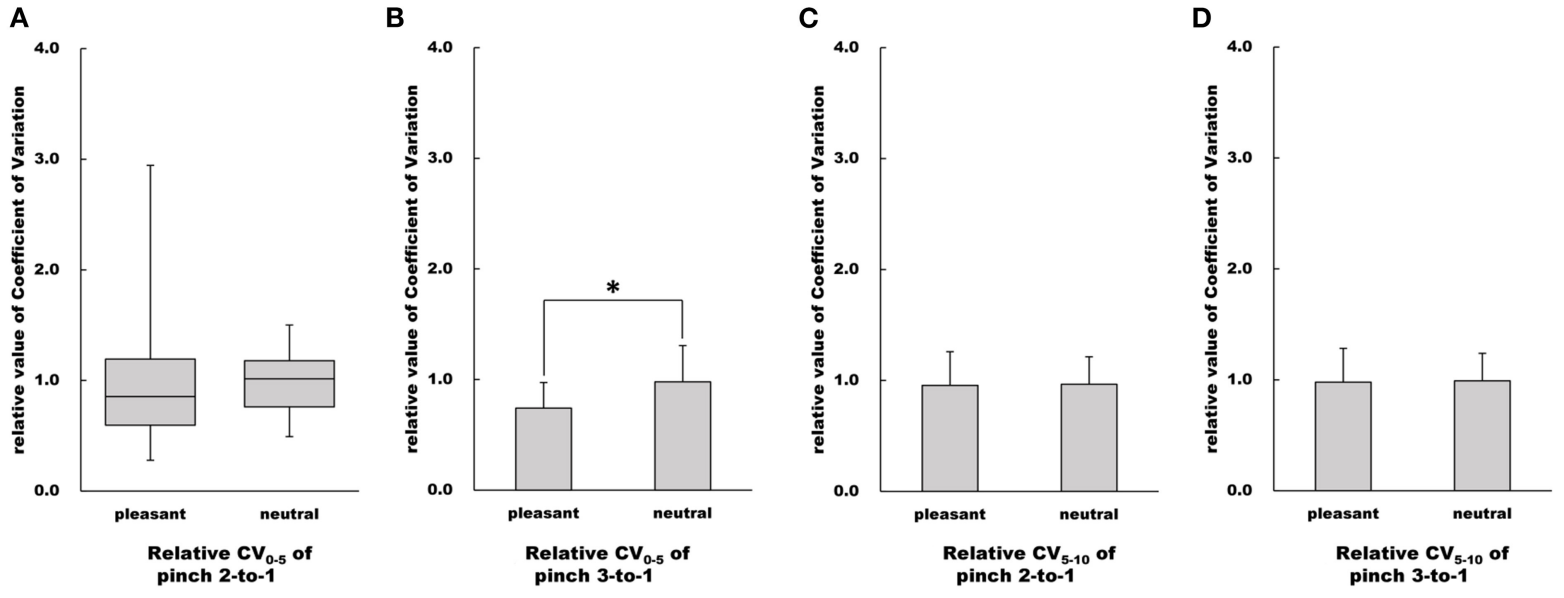
Influence of emotion on relative value of CV. **(A–D)** Show relative CV values in the pleasant and neutral image conditions, respectfully. For parametric data, relative CV values are represented as mean and standard deviation. For non-parametric data, relative CV values are represented using the box and whisker plot, with the median shown as the middle horizontal line, the upper and lower quartiles indicated as boxes, and the maximum and minimum indicated as whiskers. The vertical axis shows relative CV value, and horizontal axis shows the specific image condition (pleasant and neutral). *p < 0.05.

There was no difference in the CV_0 − 5_ value of the Pinch 2 trial relative to Pinch 1 trial between the pleasant and neutral image conditions (Mann-Whitney *U*-test, *Z* = −0.867, *p* = 0.402, *r* = 0.150, Figure 8). The CV_0 − 5_ value of the Pinch 3 trial relative to the Pinch 1 trial under the pleasant condition was significantly lower than that under the neutral image condition (Unpaired *t*-test, *t* = 2.37, 95% CI = 0.0333 to 0.443, *p* < 0.05, *r* = 0.398, Figure 8).

There were no differences in both CV_5 − 10_ values of the Pinch 2 trial relative to the Pinch 1 trial or the Pinch 3 trial relative to the Pinch 1 trial between the pleasant and neutral image conditions (Pinch 2 trial relative to the Pinch 1 trial: Unpaired *t*-test, *t* = 0.111, df = 30, 95% CI = −0.190 to 0.212, *p* = 0.912, *r* = 0.0202; Pinch 3 trial relative to the Pinch 1 trial: Unpaired *t*-test, *t* = 0.0830, df = 30, 95% CI = −0.281 to 0.305, *p* = 0.934, *r* = 0.0151, Figure 8).

**Figure 9:**
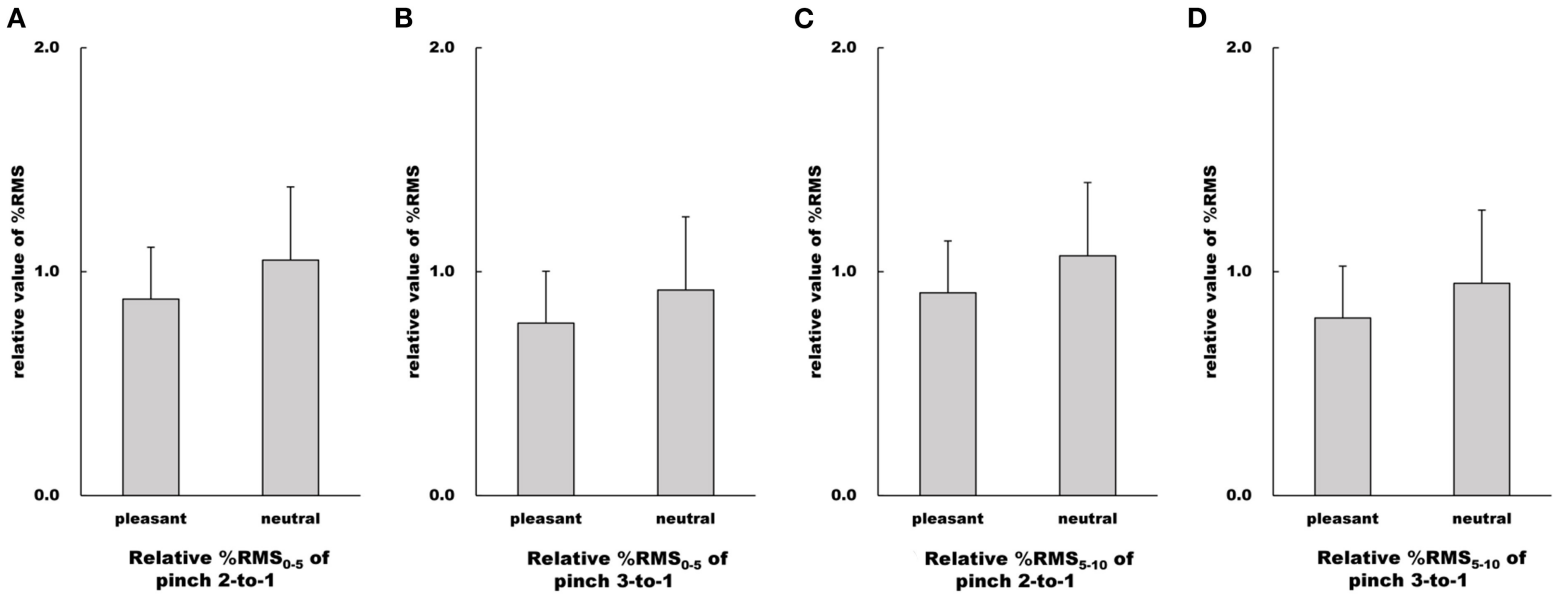
Influence of emotion on relative value of %RMS. **(A–D)** Show relative %RMS values in the pleasant and neutral image conditions, respectfully. For parametric data, relative %RMS values are represented as mean and standard deviation. For non-parametric data, relative %RMS values are represented using the box and whisker plot, with the median shown as the middle horizontal line, the upper and lower quartiles indicated as boxes, and the maximum and minimum indicated as whiskers. The vertical axis shows relative %RMS value, and horizontal axis shows the specific image condition (pleasant and neutral).

There were no differences in %RMS_0 − 5_ values of both the Pinch 2 trial relative to the Pinch 1 trial and the Pinch 3 trial relative to the Pinch 1 trial between the pleasant and neutral image conditions (Pinch 2 trial relative to the Pinch 1 trial: Unpaired *t*-test, *t* = 1.66, df = 30, 95% CI = −0.0404 to 0.388, *p* = 0.108, *r* = 0.290; Pinch 3 trial relative to the Pinch 1 trial: Unpaired *t*-test, *t* = 1.64, 95% CI = −0.0363 to 0.332, *p* = 0.112, *r* = 0.287, Figure 9).

Similarly, there were no differences in %RMS_5 − 10_ values of both the Pinch 2 trial relative to the Pinch 1 trial and the Pinch 3 trial relative to the Pinch 1 trial between the pleasant and neutral image conditions (Pinch 2 trial relative to the Pinch 1 trial: Unpaired *t*-test, *t* = 1.38, 95% CI = −0.0786 to 0.409, *p* = 0.176, *r* = 0.245; Pinch 3 trial relative to the Pinch 1 trial: Unpaired *t*-test, *t* = 1.48, 95% CI = −0.0586 to 0.369, *p* = 0.149, *r* = 0.261, Figure 9).

## Discussion

In the present study, we investigated the impact on pleasant emotion on force control during isometric precision pinch grip by non-dominant hand with visual feedback. First, the VAS score, an index of emotional valence, under the pleasant image condition was significantly higher than that under the neutral image condition. This indicates that affective visual stimuli (i.e., puppies) adopted in the present study could evoked aimed emotion (i.e., pleasant) in our subjects. Mean pinch force values under each image condition did not differ among the three pinch trials. Thus, subjects in the present study were able to exert their pinch force at 10% MVC throughout the experiment.

Regarding the results of CV_0 − 5_ value, although CV_0 − 5_ value under the neutral image condition did not differ among the three pinch trials, CV_0 − 5_ for Pinch 3 trial under the pleasant image condition was significantly lower than CV_0 − 5_ for Pinch 1 trial. In addition, relative CV_0 − 5_ pinch 3-to-1 value under the pleasant image condition was significantly lower than that under the neutral image condition. Thus, compared to neutral emotion, pleasant emotion allowed subjects to adjust pinch force to target force level more accurately using real-time visual feedback. These results differ from previous reports that have shown the emotional state did not affect force steadiness (Coombes et al., 2008; Naugle et al., 2012; Blakemore et al., 2016).

As one reason to explain the contradict results, subjects participated in previous studies performed isometric precision pinch grip task by the dominant hand (Coombes et al., 2008; Naugle et al., 2012; Blakemore et al., 2016). Because the dominant hand is found to be superior in precise motor control than the non-dominant hand (Noguchi et al., 2009; Kubota and Demura, 2011), the hand laterality may have minimized differences in CV values between affective and neutral conditions. Thus, the hand laterality may account for contradicts between the present and previous studies.

In addition, differences in attentional direction during precision pinch grip task may have influenced the result of CV value. In previous studies, precision pinch grip task and affective visual stimuli were given simultaneously. Cutaneous sensory feedback is crucial in maintaining and adapting the precise grip force control (Johansson and Cole, 1992; Augurelle et al., 2003; Monzée et al., 2003; Witney et al., 2004). Indeed, loss of cutaneous information with finger anaesthetization caused disruption of grip force control (Augurelle et al., 2003; Monzée et al., 2003). Further, as with cutaneous feedback, visual feedback should be important for precise grip force control (Slifkin et al., 2000; Vaillancourt and Russell, 2002; Christou, 2005; Sosnoff and Newell, 2006; Tracy, 2007; Baweja et al., 2009). In other words, considerable attention should be devoted to sensory-motor coordination. However, the speed and extent of information process in human brain are inherently limited (Posner et al., 1980; Marois and Ivanoff, 2005). Selective attention can be directed at particular stimuli to promote information process. Various studies have reported that attentional allocation to affective stimuli was increased (Cuthbert et al., 2000; Schupp et al., 2000, 2004). Thus, increment of attention to affective visual stimuli may have made subjects in previous studies difficult to direct their attention to sensory information during precision pinch grip task. Whereas, in the present study, precision pinch grip task was performed after subjects viewed affective picture. Consequently, precision grip force control was significantly facilitated than neutral emotion. In the present study, picture of “puppies” was utilized to evoke the pleasant emotion. Lorenz (1943) described the baby schema as a set of infantile physical features such as the large head, high and protruding forehead, large eyes, small nose and mouth, short and thick extremities and plump body shape, and so forth. These features that are commonly seen in babies and young animals promote capture attention, induce motivation and behavior for approach and caregiving. Nittono et al. (2012) and Sherman et al. (2009) have investigated the impact of cute emotion on precision motor control after viewing cute images (e.g., puppies, and kittens). Subjects participated in their studies were asked to performed a task requiring high level concentration pre-and post-picture viewing. Specifically, using tweezers, subjects were asked to remove small pieces from each hole on the patient's body depicted on the game board without touching the edges of holes. Inter-trial interval was ~4 min. After viewing cute images, the number of successful removals were significantly increased. Also, this significant effect of cute emotion on fine motor control was not observed after subjects viewed images of adult animals (Nittono et al., 2012). Thus, previous and present findings suggest that viewing young animal images allowed subjects to more pay attention to sensory information and to perform force control more precisely. As first limitation of the present study, we did not assess force steadiness using non-cute images that evoke pleasant emotion (e.g., food) or unpleasant images. In addition, we were not able to confirm whether subjects in the present study have felt cute toward picture of puppies. Thus, it may be difficult to conclude that the present result was due to a specific effect of cute emotion evoked by viewing picture of puppies.

Subsequently, regarding the results of CV_5 − 10_ value, both under the pleasant and neutral image conditions, CV_5 − 10_ values for the Pinch 1 and 2 trials were significantly lower than CV_0 − 5_ values for each trial. Greater frequency of visual feedback increased force steadiness (Slifkin et al., 2000; Sosnoff and Newell, 2005, 2006). Thus, real-time visual feedback may lead to more precise force control. In addition, both under the pleasant and neutral image conditions, CV_5 − 10_ value for the Pinch 3 trial did not differ from CV_0 − 5_ value for the Pinch 3 trial. This result indicate that repeated pinch trials promoted fine motor skill learning. Further, considering the results of CV_0 − 5_ described above, the pleasant emotion may more facilitate fine motor skill learning than the neutral emotion. However, CV_ALL_ values under both the pleasant and neutral image conditions were not observed significant differences among the three pinch trials. It may be interpreted this result as due to averaging CV_0 − 5_ and CV_5 − 10_ values.

Next, regarding the results of %RMS value, %RMS_ALL_ value for the Pinch 3 trial under the pleasant image condition was significantly lower than that for the Pinch 1 and 2 trials. Whereas, under the neutral image condition, no difference was observed among the three pinch trials. In addition, both %RMS_0 − 5_ and %RMS_5 − 10_ values for the Pinch 3 trial under the pleasant image condition was significantly lower than that for the Pinch 1 trial. Motor unit activity, such as discharge rate and synchronization, influences on force variability (Laidlaw et al., 2000; Patten and Kamen, 2000; Yao et al., 2000). Specifically, higher level of motor unit synchronization increased the amplitude of the average EMG, but not the average force. In addition, force fluctuation was increased associated with increase in motor unit synchronization (Yao et al., 2000). Indeed, increased movement accuracy reduced the amplitude of EMG (Zachry et al., 2005). Considering previous findings, as with the results of CV, reduced %RMS value of EMG recorded from left APB muscle may suggest that the pleasant emotion enabled subjects to perform more precisely pinch grip force control.

Finally, there are contradicts between the results of CV and %RMS values in the present study. First, despite that the relative CV_0 − 5_ value of the Pinch 3 trial to the Pinch 1 trial under the pleasant image condition was significantly lower than that under the neutral image condition, there was no difference in the relative %RMS_0 − 5_ value of the Pinch 3 trial to the Pinch 1 trial between the pleasant and neutral image conditions. Second, despite that the CV_5 − 10_ values of the Pinch 1 and 2 trials were significantly decreased compared to the CV_0 − 5_ values of the Pinch 1 and 2 trials under both the pleasant and neutral image conditions, significant differences were not observed for the result of %RMS value. As second limitation of the present study, although various hand muscles (e.g., APB muscle, opponens pollicis brevis muscle, flexor pollicis brevis muscle) were involved in precision pinch grip (Maier and Hepp-Reymond, 1995), the EMG activity was recorded from the APB muscle only. Thus, it is unclear to what extent the other muscles of thumb and index fingers involved in precision grip contributed to fine force control. Nevertheless, the significant reduction of the %RMS value for the Pinch 3 trial under the pleasant image condition compared to that for the Pinch 1 trial suggests that positive emotional valence enhances precision grip force control. Further research is required to resolve these contradict results of CV and %RMS values.

## Conclusion

This study investigated the effect of emotional valence on force control of precision pinch grip. Consequently, our results show that pleasant emotion can improve force control of precision pinch grip. Furthermore, our present findings suggest that therapists should consider the emotional state of patients during rehabilitation aiming to improve activities of daily living that require dexterous finger force control.

## Data availability statement

The raw data supporting the conclusions of this article will be made available by the authors, without undue reservation.

## Ethics statement

The studies involving human participants were reviewed and approved by the Research Ethics Committee at Kansai University of Health Sciences. The patients/participants provided their written informed consent to participate in this study.

## Author contributions

YB: conceptualization, methodology, investigation, data curation, formal analysis, visualization, writing-original draft, supervision, and project administration. CO: methodology, investigation, data curation, validation, writing-review and editing. All authors contributed to the article and approved the submitted version.

## Conflict of interest

The authors declare that the research was conducted in the absence of any commercial or financial relationships that could be construed as a potential conflict of interest.

## Publisher's note

All claims expressed in this article are solely those of the authors and do not necessarily represent those of their affiliated organizations, or those of the publisher, the editors and the reviewers. Any product that may be evaluated in this article, or claim that may be made by its manufacturer, is not guaranteed or endorsed by the publisher.
